# A Text-Book on Diseases of the Eye

**Published:** 1890-06

**Authors:** 


**A Text-Book on Diseases of the Eye.** By HENRY D. NOYES, A.M., M.D., Professor of Ophthalmology and Otology in Bellevue Hospital Medical College; Executive Surgeon to the N. Y. Eye and Ear Infirmary; recently President of the American Ophthalmological Society, etc., etc. Royal octavo, 733 pages, richly illustrated with chromo-lithographic plates and 226 engravings. Price, bound in extra muslin, $6.00; in sheep, $7.00. New York: William Wood Company.

The appearance of a text-book on ophthalmology by an American author must be hailed with welcome, especially when coming from so high a source as Prof. Noyes. The book is intended to be practical rather than theoretical, and herein Prof. Noyes is but following the tendency of the times. This volume is an outgrowth of the author's treatise on "Diseases of the Eye," published in Wood's Library of Standard Medical Authors. It follows the same general order of arrangement, viz.: the first part devoted to the general anatomy and physiology of the eye, with its functional disorders; the second part treating of inflammation and organic textural changes.

The spirit of the book is clinical, but an adequate preparation for clinical and practical work means a thorough and comprehensive preliminary knowledge. To make the book as practical as possible, mathematical formulæ have been omitted, and herein Prof. Noyes is to be congratulated. We doubt whether in a treatise on ophthalmology intended for students and practitioners, a deep study of obtuse laws of optics and trigonometrical functions is in good taste, certainly not when some of the preparatory courses of its readers are considered.

The pathology and microscopic anatomy of some of the organic lesions have been presented in such a way as to give an intelligent and ample account of these morbid processes. The relations of micro-organisms as exciting causes in the production of diseases of the eye have been fully recognized. No little labor has been spent in setting forth the relations of the eye to the brain and nervous system. Most ophthalmic text-books pass over this chapter with a few desultory remarks, leaving the matter to text-books on neurology, whereas these point to works on ophthalmology for the required information. Prof. Noyes has not shirked his duty in this respect, as both illustrations and descriptions will testify.

The author has not only quoted his own cases and experiences, but has also familiarized himself with the work and experience of others, and given credit wherever and whenever due. The references and bibliography show him to be abreast of his subject at home and abroad.

On the whole, we must say in justice to ourselves, to the author, and to the publisher, that it is the best, neatest and cleanest text-book on ophthalmology that has been our good fortune to examine.

